# Primary Epithelioid Sarcoma of the Scalp Complicated by Humoral Hypercalcemia of Malignancy

**Published:** 2012-12-10

**Authors:** Ilaria Tocco, Franco Bassetto, Vincenzo Vindigni

**Affiliations:** Clinic of Plastic Surgery, University of Padova, Italy

**Figure:**
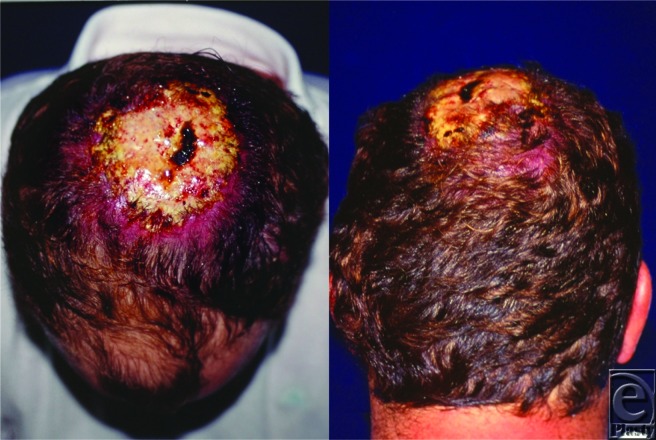


## DESCRIPTION

A 32-year-old man was referred to the Clinic of Plastic Surgery for evaluation of an enlarging, nonhealing lesion on the scalp that had been present for 12 months. The patient was somnolent and dehydrated. Serum calcium level was 4.5 mmol/L (normal 2.1–2.6 mmol/L).

Incisional biopsies from 4 quadrants demonstrated an epithelioid sarcoma comprising epithelioid cells and discrete areas of central necrosis. Plain skull radiographs showed bony erosion. Full-body computed tomographic scans confirmed a soft-tissue mass fixed to the skull and multiple metastatic nodules in the cervical lymph nodes and both lungs.

## QUESTIONS

1. **What are the characteristics of epithelioid sarcoma?**
2. **What is the treatment plan for an epithelioid sarcoma of the scalp?**
3. **What are the pathogenesis and treatment of humoral hypercalcemia of malignancy?**

## DISCUSSION

Epithelioid sarcoma is a mesenchymal malignancy that has been mostly reported as affecting the distal extremities. It is exceptionally rare as a primary neoplasm in the head and neck. Early diagnosis poses a challenge. The first appearance of the tumor can be very ambiguous, most frequently as slow-growing subcutaneous nodules and ulcers that are easily misdiagnosed as benign lesions for long times, as was the reported case. Moreover, even the histological diagnosis may pose some difficulties. Specific enzymatic, histochemical, and ultrastructural tests are required for accurate diagnosis.

A treatment plan for this neoplasm has not been well defined in the literature because of the rarity of sarcomas in general and of epithelioid sarcomas located in the head and neck in particular. The tumor may be localized in the dermis and subcutaneous tissue, but it has a propensity for extension through deeper structures, even the cortical bone. Thus, it is imperative that antitumor therapy be implemented promptly. Early intervention is important. For epithelioid sarcoma, aggressive surgical management and radical lymphadenectomy seem to be the treatments of choice. Reconstruction of the tumor excision defect can be challenging. Chemotherapy may play a limited role in advanced stages. Radiation therapy, although usually performed, needs further research on efficacy.

The osteolytic process of bone invasion and several factors generated by disseminated tumor (such as parathyroid hormone–related peptide) can result in an increase in serum calcium concentration, a condition known as humoral hypercalcemia of malignancy. Hypercalcemia can potentially threaten the life of the patient, and this previously unreported association with epithelioid sarcoma stresses the importance of a high index of suspicion for early diagnosis. Treatment of tumor-related hypercalcemia consists of normal saline administered intravenously at a rate of 200 to 500 mL per hour, followed by intravenous bisphosphonate. If normocalcemia is not achieved, the prognosis is very poor, and approximately 50% of such patients die within 30 days.

## References

[1] Varela JM, Valencia J, Jimenez F, Torres A (1989). Epithelioid sarcoma. Eur J Plast Surg.

[2] Tran LM, Mark RM, Meier R, Calcaterra CT, Parker RG (1992). Sarcomas of the head and neck. Cancer.

[3] Suwantemee C (1999). Primary epithelioid sarcoma of the scalp. Plast Reconstr Surg.

[4] Bos GD, Pritchard DJ, Reiman HM, Dobyns JH, Ilstrup DM, Landon GC (1988). Epithelioid sarcoma: an analysis of fifty-one cases. J Bone Joint Surg Am.

[5] Phillips T, Pollock D, Sommerland B, Baker H (1986). Epithelioid sarcoma—a case report. Clin Exp Dermatol.

[6] Body JJ, Dumon JC (1994). Treatment of tumor-induced hypercalcaemia with the bisphosphonate pamidronate: dose-response relationship and influence of the tumor type. Ann Oncol.

[7] Stewart AF (2005). Hypercalcemia associated with cancer. N Engl J Med.

[8] Chase DR, Enzinger FM (1985). Epithelioid sarcoma: diagnosis, prognostic indicators and treatment. Am J Surg Pathol.

